# Association of submaximal exercise test variables with diagnosis of asthma in a mixed-breed cohort of leisure horses

**DOI:** 10.1093/jvimsj/aalag227

**Published:** 2026-09-22

**Authors:** Lia K Meiseberg, Julien Delarocque, Nicole de Buhr, Bernhard Ohnesorge

**Affiliations:** Clinic for Horses, University of Veterinary Medicine, Bünteweg 9, 30559 Hannover, Germany; Institute of Biochemistry, University of Veterinary Medicine, Bünteweg 17, 30559 Hannover, Germany; Clinic for Horses, University of Veterinary Medicine, Bünteweg 9, 30559 Hannover, Germany; Nancy Fair Link Laminitis Research Center, Department of Comparative Biomedical Sciences, College of Veterinary Medicine, Mississippi State University, 240 Wise Center Drive, 39762 Mississippi State, United States; Institute of Biochemistry, University of Veterinary Medicine, Bünteweg 17, 30559 Hannover, Germany; Research Center for Emerging Infections and Zoonoses (RIZ), University of Veterinary Medicine, Bünteweg 17, 30559 Hannover, Germany; Clinic for Horses, University of Veterinary Medicine, Bünteweg 9, 30559 Hannover, Germany

**Keywords:** arterial blood gas, chronic obstructive bronchitis, equine asthma phenotypes, inflammatory airway disease, recovery, exercise

## Abstract

**Background:**

Submaximal exercise tests (SET) are used to assess respiratory health in various contexts. The diagnostic value of SETs for the assessment of asthma in the field remains uncertain.

**Hypothesis/Objectives:**

A SET helps distinguish healthy horses and asthma phenotypes.

**Animals:**

Twenty-six privately owned mixed-breed leisure horses of a clinical cohort. Horses were healthy (8) or diagnosed with mild (miEA) (4), moderate (modEA) (7), or severe asthma (sEA) (7) according to history, clinical examination, and bronchoalveolar lavage fluid (BALF) cytology.

**Methods:**

Cross-sectional study using clinical data and samples from a hospital cohort. The SET included ECG, arterial blood gas, lactate, respiratory rate (RR), and heart rate (HR) analysis. Samples were taken at rest (time point (T) 0), and after defined intervals (twice 2 minutes trot (T1, T2) and gallop (T3, T4), followed by 3 minutes trot (T5) and gallop (T6)). HR and RR recovery were evaluated. Predictive values of variables towards diagnosis of asthma were estimated with ordinal models.

**Results:**

Arterial partial pressure of oxygen (pO_2_) at T0 had the strongest association with diagnosis (τ = −.513, LRT = 18.1, adj. *P* < .001). Hypoxemia at T6 was evident in all healthy and miEA horses. In contrast, some horses with modEA and sEA presented with increased pO_2_ at T6 compared to T0. Recovery lengths had no significant association with diagnosis (RR τ = .112, LRT = .805, adj. *P* = .493; HR τ = .174, LRT = 1.528, adj. *P* = .287) and demonstrated wide intra-group variation.

**Conclusions and clinical importance:**

The SET did neither confirm diagnosis of asthma nor provide adequate results to assess respiratory health or disease in this cohort.

## Introduction

Asthma in horses is a chronic, non-infectious inflammatory disease of the lung that affects over 80% of some horse populations.^1^^,^^2^ Clinical features include airway inflammation and hyperreactivity, excessive mucus production, and bronchospasm,^3^ manifesting clinically as exercise intolerance, cough, and dyspnea.^4^ The disease is understood as a continuum, with clinical pictures ranging from subclinical (mild) to moderate to severe cases, with some degree of overlap.^4^^,^^5^ Lower airway inflammation contributes to poor performance in equine athletes.^6–9^ In mild EA (miEA), exercise intolerance is considered the most reliable divergent clinical variable,^10^^,^^11^ since additional clinical signs are absent, and non-invasive biomarkers are lacking.^11^

Submaximal exercise tests (SETs) can be employed in examination protocols used in the assessment of a horses’ health and fitness.^12^ One example is the use of SETs during pre-purchase evaluations.^13^ SETs can also be a component of respiratory, cardiac, or musculoskeletal workups for investigating the cause of poor performance.^7^^,^^14^^,^^15^ The assessment of exercise intolerance in horses of mixed breeds with low performance levels can be challenging. In a field setting, the evaluation is influenced by subjective assessments from the owner or rider, as well as by internal and external factors.^16^^,^^17^ Individual factors are the level of training, the body condition score (BCS), general health, or even excitement during the test.^18–20^ Moreover, the general knowledge is based on SETs conducted on homogeneous cohorts of sport horses.^21^ Examples are treadmill tests on thoroughbred racehorses or standardized length examinations on endurance horses.^10^^,^^22^^,^^23^ Anecdotal evidence suggests that the length of the recovery time, defined as the time required to attain resting values of heart rate (HR) and respiratory rate (RR), might serve as a practical indicator for assessing the horses’ health in a field setting.^24^ However, studies and clear-defined reference intervals are lacking, although the test is used in practice^21^^,^^25^ and deemed to be of forensic value in diagnosing “heaves” in Germany.^26^

The objective of this study was to retrospectively assess the diagnostic accuracy of variables of a SET for the practical evaluation of defined asthma phenotypes (miEA, moderate (modEA) and sEA) in a cohort of non-athlete horses that were presented to the clinic for respiratory or general health workups. We hypothesized that RR, HR, and recovery times would increase with disease severity during the SET, while exercise would exacerbate differences in blood lactate concentrations and arterial partial pressure of oxygen (pO_2_).

## Materials and methods

### Animals

Twenty-six privately owned mixed-breed leisure horses were diagnosed as healthy, mild, moderate, or severe asthmatic through respiratory workup using a standardized diagnostic protocol at the Clinic for Horses of the University of Veterinary Medicine Hannover, between December 2021 and March 2023. Individual diagnoses were based on medical history, clinical examinations, and bronchoalveolar lavage fluid (BALF) cytology results as published elsewhere.^5^

### Study design

**Figure 1:**
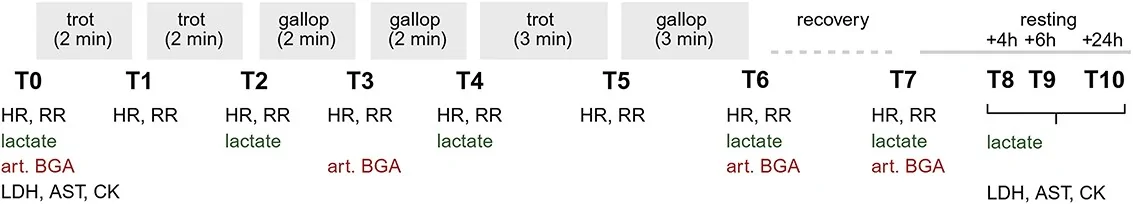
Graphical illustration of the SET protocol. The top row indicates the different phases of the SET. The gait and duration were pre-defined for the exercising phases (gray boxes). Spaces between the boxes indicate brief interruptions at the pre-defined time points (T). The length of the recovery period was defined by the horses’ individual recovery of HR and RR (indicated as dashed line). Recovery was followed by stall rest with sampling at 4, 6, and 24 hours post T7 (indicated as solid gray line). The variables taken at specific T are indicated below the individual T. Abbreviations: T = time point; HR = heart rate; RR = respiratory rate; L = lactate; art. BGA = arterial blood gas analysis; LDH = lactate dehydrogenase; AST = aspartate aminotransferase; CK, creatine kinase. Created under license with biorender.com.

A standardized examination protocol including the SET was conducted on a clinical cohort as health check-up, and SET data retrospectively analyzed for their association with diagnosis of asthma. Details are included in the Appendix. Briefly, all horses underwent an unridden SET on a lunge in an indoor riding arena on a circle with a diameter of approximately 14 m. The SET was conducted under the supervision of the same veterinarian examining, with 2 assistants. During the SET, exercise was stopped briefly at defined intervals to measure the HR and RR via auscultation, and for blood sampling for arterial blood gas and lactate analysis (Figure 1). Arterial and venous catheters were placed before the SET. An electrocardiogram (ECG) (Televet®, Engel Engineering Services GmbH, Heusenstamm, Germany), starting a minimum of 2 hours before the SET, was evaluated using the Televet® software (version 7.0.0.0) after the SET to rule out clinically significant cardiac disease.

The recovery time was defined as the interval between T6 and return to physiological resting HR (up to 40 beats per minute (bpm)) and RR (up to 18 respirations per minute (rpm)) (T7). For asthmatic horses, the individual resting values were used as they exceeded physiological values in some cases. The length of the recovery time was recorded separately for HR and RR, using the continuous ECG readings for the former and counts performed every minute for RR. Electrocardiogram recordings were used during recovery to impede excitement from auscultation. All horses were led for the first 5 minutes and then came to a halt for the rest of the recovery period. Electrocardiogram monitoring was terminated after T7. Muscle enzymes were measured initially and 4-, 6-, and 24-hours post exercise. Horses remained in hospital for at least 24 hours after the exercise test and were examined for general health before discharge.

### Statistical analysis

Statistical analysis was performed with R 4.5.^27^ Details are presented in the Appendix. Briefly, the predictive value of clinical and laboratory variables toward the diagnosis of asthma was estimated with ordinal models. The *P*-values were adjusted for multiple comparisons. Correlations between variables were described with Spearman’s rho. Factor analysis of mixed data was used to obtain a unified depiction of all variables.

## Results

### Feasibility of the procedure

A total of 25 horses successfully completed the SET. In 1 horse (ID5), the test was shortened by the supervising veterinarian during the final gallop phase due to evident respiratory distress and fatigue. This horse was classified as severe EA (sEA) and exhibited the most severe clinical signs and the most profound resting global insufficiency of the lung among all study horses (pO_2_ of 67 mmHg and pCO_2_ of 54.2 mmHg). All horses were accustomed to lunging and came to a halt within a half to 1 circle length depending on pace. Arterial catheter placement was successful in 17/26 horses. In 5/9 unsuccessful cases, the attempt was aborted due to defensive behavior with the nose twitch. In the remaining 3/9 cases, placement was successful, but exercising resulted in a loss of function. The malfunction or missing of the catheter resulted in the omission of blood gas values at T3 in 10/26 horses and in 2/26 at T6 and T7.

### Exclusion of cardiovascular and muscular disease

No indications of cardiovascular disease were observed in any horse in the clinical examination. Analysis of the ECG recordings revealed the presence of arrhythmia at rest in 5 horses (19.23%). The observed arrhythmias were single premature atrial complexes (in 2 horses), single premature ventricular complexes (in 5 horses), and paroxysmal ventricular tachycardia with 2 phases resolving after approximately 1 minute (in 1 horse). No ECG abnormalities were observed during exercise or during recovery in any of the horses. Furthermore, no clinical indications of muscular disease were observed in any horse. The resting and post-exercise measurements of the muscle enzymes (AST, LDH, CK) revealed no elevations above the reference ranges except for 1 horse after 24 hours (AST: 1224 U/L, moderate EA, ID18) (Supplementary File 1). There were no clinical indications of muscular disease at that time, so no further diagnostic procedures were performed.

### Association of SET variables with diagnosis at single time points

**Figure 2:**
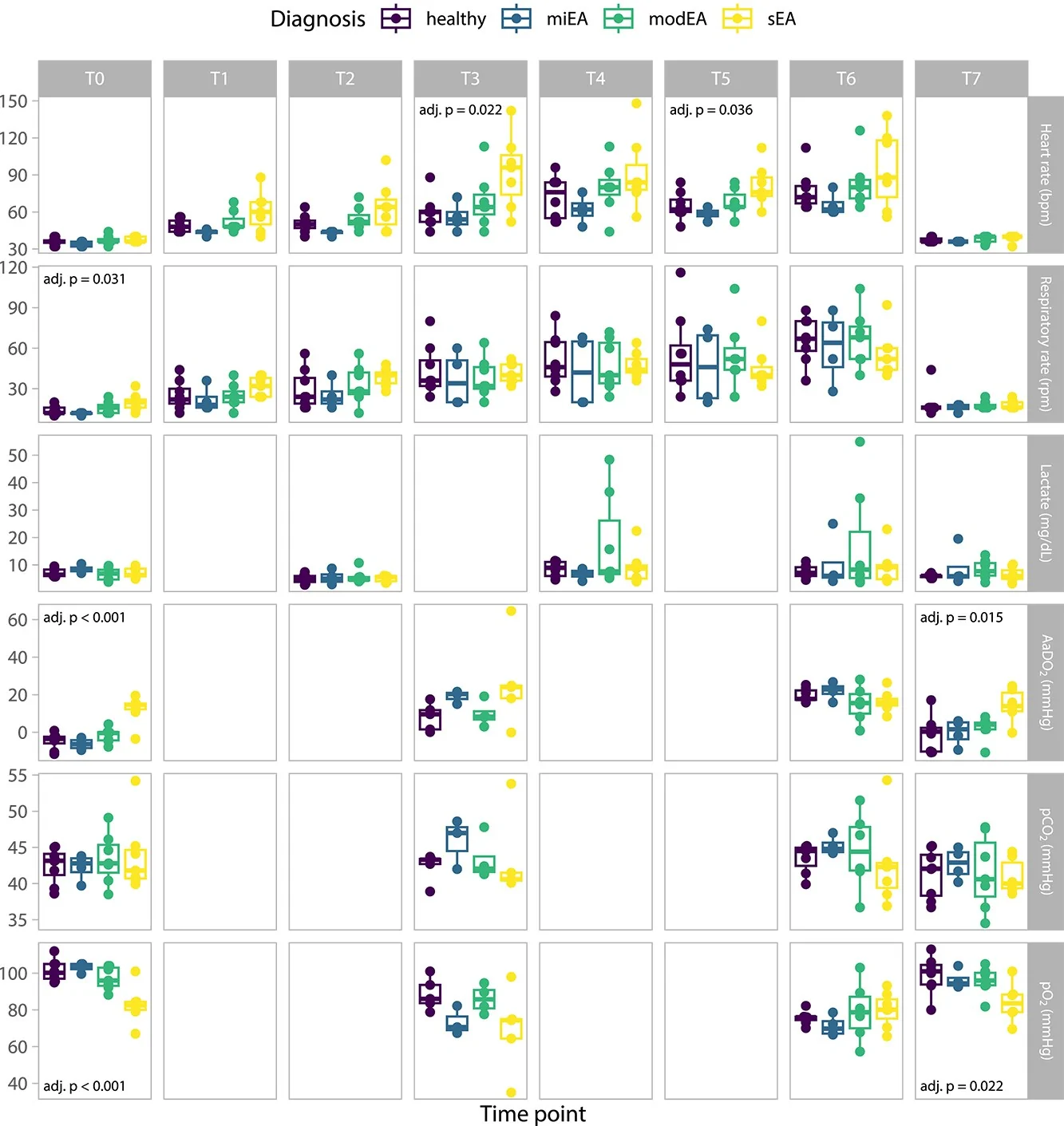
Comparisons of variables at different time points of the SET and association with diagnosis. The time point (T) is shown in the first row with all associated measures presented vertically underneath. The measures of a specific variable are presented in a square box. The depictured variables and units are shown on the right. Individual ranges of measures are shown on the left. Data are presented as median and quartiles; each dot represents 1 horse. Significant associations between variable values and diagnosis at a specific T are shown within the corresponding square. Abbreviations: AaDO_2_ = alveolar-arterial oxygen gradient; miEA = mild EA; modEA = moderate EA; pCO_2_ = arterial carbon dioxide partial pressure; pO_2_ = arterial oxygen partial pressure; sEA = severe EA.

Figure 2 provides an overview of the association between diagnosis and clinical variables over time. The associated data (median, lower and upper quartiles) are further presented in Supplementary File 2. With the use of an ordinal model utilizing diagnosis as dependent variable, single SET variables were significantly associated with diagnosis: HR at T3 (adj. *P* = .022) and T5 (adj. *P* = .036), and RR at rest (T0, adj. *P* = .031). In addition to that, significant associations of the diagnosis with pO_2_ and AaDO_2_ were observed at T0 (both adj. *P* < .001) and T7 (adj. *P* = .022, adj. *P* = .015, respectively). However, variance between individuals was large, resulting in overlapping variable values between groups. All other tested variables or time points were not significantly associated with diagnosis (Table 1). A Spearman correlation matrix for all variables is displayed in Supplementary File 3.

**Table 1:** Association of SET variables with diagnosis.

**Time point**	**Df**	**AIC**	**LRT**	***P***	**adj. *P***	**OR [95% CI]**	**τ_B_ [95% CI]**
**Heart rate**							
0	1.000	76.574	1.882	.170	.330	1.169 [0.936; 1.490]	0.245 [−0.042; 0.533]
1	1.000	76.574	5.892	.015	.056	1.095 [1.017; 1.200]	0.260 [−0.068; 0.589]
2	1.000	76.574	5.882	.015	.056	1.079 [1.014; 1.170]	0.292 [−0.015; 0.599]
3	1.000	76.574	8.727	.003	**.022**	1.053 [1.016; 1.100]	0.379 [0.122; 0.636]
4	1.000	76.574	3.892	.049	.146	1.034 [1.000; 1.075]	0.221 [−0.078; 0.521]
5	1.000	76.574	7.103	.008	**.036**	1.084 [1.020; 1.169]	0.363 [0.084; 0.642]
6	1.000	76.574	3.426	.064	.163	1.032 [0.998; 1.073]	0.206 [−0.123; 0.536]
7	1.000	76.574	2.520	.112	.259	1.294 [0.944; 1.853]	0.306 [−0.049; 0.661]
**Respiratory rate**							
0	1.000	76.574	7.687	.006	**.031**	1.250 [1.063; 1.533]	0.415 [0.159; 0.671]
1	1.000	76.574	1.935	.164	.330	1.058 [0.978; 1.151]	0.257 [−0.036; 0.550]
2	1.000	76.574	2.444	.118	.259	1.047 [0.989; 1.116]	0.289 [0.009; 0.568]
3	1.000	76.574	0.058	.810	.872	0.994 [0.949; 1.043]	0.041 [−0.218; 0.299]
4	1.000	76.574	0.111	.739	.872	0.994 [0.955; 1.033]	−0.022 [−0.253; 0.210]
5	1.000	76.574	0.379	.538	.806	0.991 [0.961; 1.020]	−0.083 [−0.374; 0.208]
6	1.000	76.574	0.711	.399	.627	0.985 [0.947; 1.020]	−0.156 [−0.441; 0.129]
7	1.000	76.574	0.031	.859	.872	0.988 [0.860; 1.157]	0.234 [−0.075; 0.542]
**Lactate**							
0	1.000	76.574	0.095	.758	.872	0.596 [0.021; 16.824]	−0.028 [−0.303; 0.246]
2	1.000	76.574	0.039	.844	.872	1.385 [0.048; 39.417]	0.057 [−0.259; 0.372]
4	1.000	76.574	0.317	.573	.806	1.167 [0.669; 2.076]	−0.032 [−0.334; 0.270]
6	1.000	76.574	0.297	.586	.806	1.141 [0.698; 1.903]	0.032 [−0.221; 0.284]
7	1.000	76.574	0.098	.754	.872	1.294 [0.262; 7.813]	0.014 [−0.259; 0.287]
**pCO**_**2**_							
0	1.000	76.574	0.821	.365	.602	1.104 [0.887; 1.386]	0.049 [−0.259; 0.357]
3	1.000	52.458	0.026	.872	.872	1.020 [0.787; 1.304]	−0.240 [−0.684; 0.203]
6	1.000	71.470	0.237	.626	.827	0.956 [0.785; 1.146]	−0.124 [−0.434; 0.187]
7	1.000	73.839	0.059	.808	.872	0.976 [0.798; 1.189]	−0.057 [−0.344; 0.230]
**pO**_**2**_							
0	1.000	76.574	18.102	.000	**.000**	0.812 [0.708; 0.904]	−0.513 [−0.762; 0.263]
3	1.000	52.458	3.656	.056	.154	0.936 [0.854; 1.002]	−0.323 [−0.712; 0.065]
6	1.000	71.470	1.084	.298	.517	1.037 [0.969; 1.118]	0.177 [−0.096; 0.451]
7	1.000	73.839	8.595	.003	**.022**	0.881 [0.793; 0.961]	−0.390 [−0.684; −0.095]
**AaDO**_**2**_							
0	1.000	76.574	20.769	.000	**.000**	1.305 [1.146; 1.562]	0.493 [0.246; 0.740]
3	1.000	52.458	4.003	.045	.146	1.084 [1.001; 1.220]	0.290 [−0.114; 0.695]
6	1.000	71.470	1.595	.207	.379	0.934 [0.830; 1.038]	−0.231 [−0.463; 0.001]
7	1.000	73.839	10.228	.001	**.015**	1.158 [1.055; 1.301]	0.442 [0.155; 0.729]

The results of the ordinal models are presented for time points 0, 3, 6, and 7 in association with the diagnosis. Significant overall values are presented in bold. The diagnosis showed the strongest negative association with pO_2_ at rest (T0). The association is described with Kendall’s correlation coefficient τ_B_ and odds-ratios with 95% CIs. *P*-values were adjusted across all models with the Benjamini-Hochberg method. Abbreviations: AaDO_2_ = alveolar-arterial oxygen gradient; CI = confidence interval; pO_2_ = arterial oxygen partial pressure; pCO_2_ = arterial carbon dioxide partial pressure; OR = odds-ratio.

**Figure 3:**
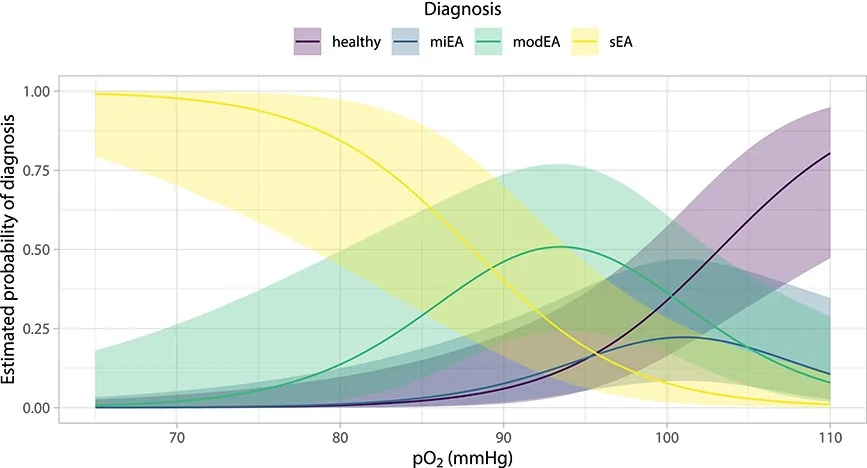
Estimated probability of each diagnosis depending on basal pO_2_. The graph shows the predicted probability of the different diagnoses and their 95% CI (y-axis) at different values for basal pO_2_ (x-axis). It indicates an overall wide overlap of diagnoses in horses presented with physiological arterial blood gas values (85-110 mmHg), presented as overlapping of colored curves. Values underneath 80 mmHg predict sEA with a probability of nearly 90% (yellow curve). Abbreviations: miEA = mild EA; modEA = moderate EA; pO_2_ = arterial oxygen partial pressure; sEA = severe EA.

Basal pO_2_ showed the strongest association with diagnosis (Tau = −.513, adj. *P* < .001). The predicted probability of each diagnosis is presented in Figure 3. There is significant overlap between the most likely diagnoses in the 85-110 mmHg range, leading to poor overall diagnostic accuracy (50% [29.9; 70.1%]). However, sEA could be distinguished from all other categories with 85.7% sensitivity and 94.7% specificity because of the lower basal pO_2_ (<85 mmHg) values generally associated with this diagnosis. The model’s positive predictive value for sEA was 85.7%, and its negative predictive value 94.7% in this cohort. Data for pO_2_ and pCO_2_ for all time points are presented in Supplementary File 4.

### Evaluation of maximum and minimum values

We further investigated the maximum values for HR and RR, pCO_2_ and AaDO_2_, in addition to the minimum value of pO_2_ throughout all time points as predictors for diagnosis with an ordinal model. None of the tested maximum values showed significant associations with the diagnosis after correcting for multiple comparisons (Table 2). Median (range) for healthy was 84 bpm (64-112), for mild 64 bpm (60-80), for moderate 80 bpm (64-126), and for sEA 100 bpm (72-148). On the contrary, median maximum RR decreased in sEA. The median (range) for healthy was 67 rpm (36-116), for mild 64 rpm (28-88), for moderate 72 rpm (40-104), and for sEA 56 rpm (44-92).

**Table 2:** Associations of the maximum (HR, RR, lactate, pCO_2_, and AaDO_2_) or minimum (pO_2_) values with diagnosis.

	**Df**	**AIC**	**LRT**	***P***	**adj. *P***	**OR [95% CI]**	**τ** _**B**_ **[95% CI]**
**pO**_**2**_	1.000	76.574	1.197	.274	.548	0.970 [0.915; 1.025]	−0.060 [−0.337; 0.217]
**Lactate**	1.000	76.574	0.196	.658	.687	1.107 [0.693; 1.776]	−0.063 [−0.334; 0.207]
**pCO**_**2**_	1.000	76.574	0.163	.687	.687	1.042 [0.847; 1.280]	−0.060 [−0.352; 0.232]
**AaDO**_**2**_	1.000	76.574	1.460	.227	.548	1.041 [0.975; 1.126]	0.088 [−0.194; 0.370]
**HR**	1.000	76.574	4.296	.038	.229	1.038 [1.002; 1.081]	0.261 [−0.013; 0.534]
**RR**	1.000	76.574	0.742	.389	.584	0.985 [0.951; 1.019]	−0.133 [−0.410; 0.143]

Results of the ordinal model show no significant associations of any of the maximum values investigated with the diagnosis of asthma in this study cohort. The association is described with Kendall’s correlation coefficient τ_B_ and odds-ratios with 95% CIs. *P*-values were adjusted with the Benjamini-Hochberg method. Abbreviations: AaDO_2_ = alveolar-arterial oxygen gradient; CI = confidence interval; pO_2_ = arterial oxygen partial pressure; pCO_2_ = arterial carbon dioxide partial pressure; HR = heart rate; RR = respiratory rate; OR = odds-ratio

### Diagnostic value of the duration of HR and RR recovery

**Figure 4:**
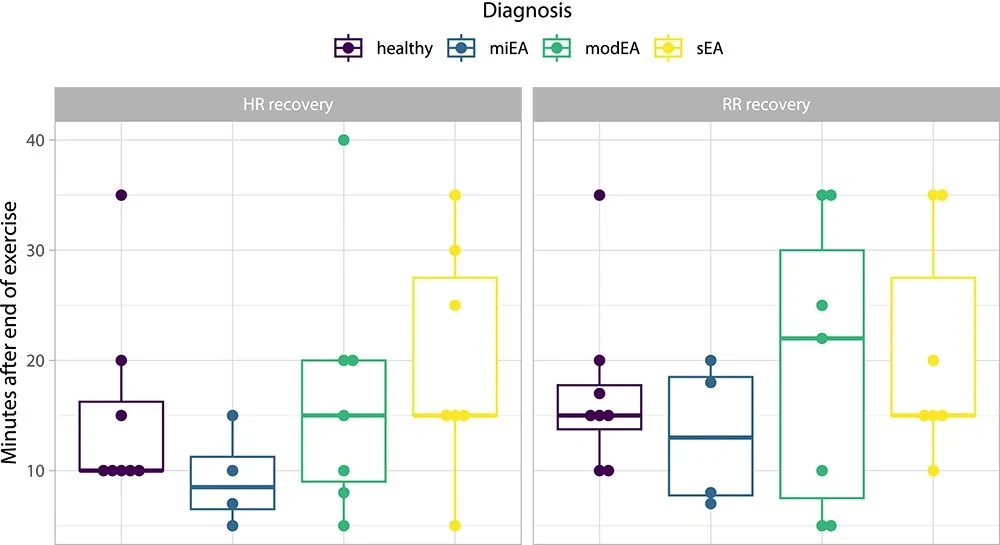
Duration of heart rate (HR) and respiratory rate (RR) recovery between the phenotypes. Length of the HR recovery of the phenotypes is presented on the left, whereas RR recovery length is presented on the right. Large inter-individual variations were observed within the phenotypes, which increased with disease severity. Data is presented as median and quartiles. The visible single outliers in the healthy (HR, RR) and moderate EA (HR) phenotype were anxious Icelandic horses. Abbreviations: miEA = mild EA; modEA = moderate EA; sEA = severe EA.

There was no significant association of the lengths of the recovery time of HR and RR with diagnosis (RR τ = .112, LRT = .805, adj. *P* = .493; HR τ = .174, LRT = 1.528, adj. *P* = .287). We observed large inter-individual variation within the groups (Figure 4). Median value (range) of HR recovery was 10 minutes (10-35) for healthy, 8.5 minutes (5-15) for mild, 15 minutes (5-40) for moderate, and 15 minutes (5-35) for sEA. Median (range) of RR recovery was 15 minutes (10-35) for healthy, 13 minutes (7-20) for mild, 22 minutes (5-35) for moderate, and 15 minutes (10-35) for sEA. Overall, recovery times for both variables were widely overlapping between the phenotypes.

### Change of pO_2_ over recovery

**Figure 5:**
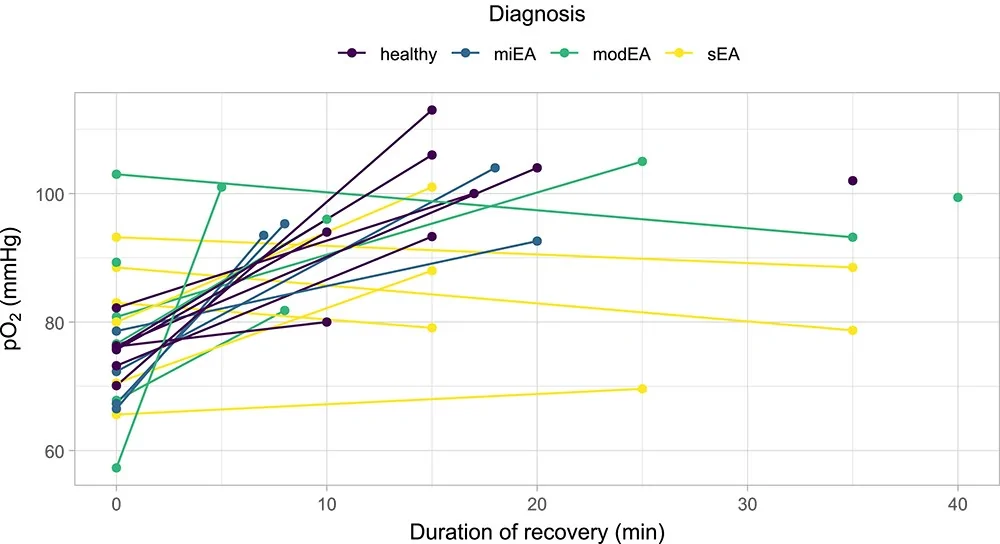
Differences of pO_2_ during recovery. Changes in pO_2_ from end of exercise (T6, indicated as minute 0 of the recovery time on the x-axis, first dot) and after reaching the individual HR and RR recovery (T7, second dot) are presented. Connected data points indicate values of the same individual horse. Single values at T7 are present since sampling at T6 was not successful in those horses. Clinically non-asthmatic horses (healthy and miEA) presented with exercise-induced hypoxemia, followed by an increase of pO_2_ during recovery, whereas the effect of recovery on pO_2_ in horses with modEA and sEA was diverse. Phenotypes are color-coded as presented above the graph. Abbreviations: miEA = mild EA; modEA = moderate EA; pO_2_ = arterial oxygen partial pressure; sEA = severe EA.

Arterial blood gases were obtained directly after exercise (T6, 0 minutes of recovery) and at the end of the individual recovery time (T7). Differences in pO_2_ from T6 to T7 are presented in Figure 5. Horses classified as healthy and miEA presented with exercise-induced hypoxemia, which resolved closer to baseline values during recovery. Horses classified as moderate and sEA showed diverse effects of exercise, as some horses presented with increased pO_2_ compared to T0 (Supplementary File 4) and decreasing pO_2_ over recovery.

### Heterogeneity of the study cohort during SET

**Figure 6:**
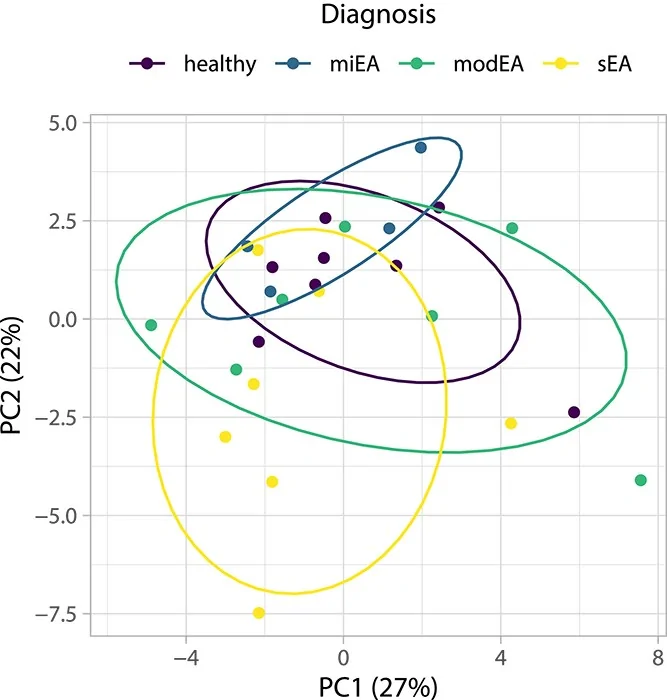
Factor analysis of mixed data on the overall SET data. The clinical presentation of the 3 asthma phenotypes in comparison to lung healthy controls is visualized. Every point embodies a single horse. The colors indicate the diagnosis, which was not included in the analysis. A 68% confidence ellipse is shown for each group. This analysis indicates that the clinical presentations of all 3 phenotypes and healthy horses considerably overlap while performing a SET, and heterogeneity increases with disease severity. Abbreviations: PC = principal component; miEA = mild EA; modEA = moderate EA; sEA = severe EA.

Factor analysis of the consolidated dataset reveals marked overlapping of the asthma phenotypes and an increasing phenotypical variability during exercise with disease severity in this cohort (Figure 6).

## Discussion

This study aimed to assess the predictive value of clinical and laboratory variables obtained during a SET for the diagnosis of asthma. External factors, such as the weather on the day of examination, and internal factors, such as the BCS and training levels, were not accounted for to reflect field conditions of practitioners examining mixed cohorts.

### Clinical variables during the SET and recovery

It is established that inflammation of the lower respiratory tract can affect performance in equine athletes through several mechanisms,^28–30^ but it is unclear whether this is the case in non-athlete horses under field conditions.^31^ While there is a higher likelihood of diagnosing inflammatory airway disease (IAD) in poorly racing Standardbreds,^32^ this describes an extreme situation with highly standardized performance expectations. Different conclusions were reached in other cohorts such as well-performing show jumping and dressage horses, whose performance was not markedly affected by IAD.^33^ Unfortunately, there are fewer studies investigating the effect of exercise on horses suffering from severe asthma (formerly “chronic obstructive bronchitis” or “heaves”).^34–37^

In the present study on a mixed cohort of leisure horses, clinical variables such as HR and RR were associated with diagnosis at some time points only (Figure 2). HR specifically had higher correlations with diagnosis within and towards the end of the SET (T3, T5). On the contrary, RR had essentially no correlation with diagnosis during the exercise and was most helpful at rest (T0). Finally, these variables had large overlaps between phenotypes, resulting in limited diagnostic usefulness, independently of statistical significance. Given that RR was different between diagnoses at rest, but not during exercise or recovery, it appears likely that horses with sEA do not increase their RR beyond a certain level during exercise. This could be due to the required effort, the fact that a higher RR becomes less efficient because of bottlenecks in, for example, the inspiratory capacity or the ventilatory reserve volume, as seen in human asthmatics, or a combination of these modalities.^38^^,^^39^ These differences are possibly attributable to variances of the gas flow through the respiratory system or to changes in gas exchange, possibly due to an increased diffusion barrier and higher resistance in the inflamed airways.^40^ We therefore conclude that more strenuous exercise would probably not have improved the diagnostic accuracy of the RR during the SET between diagnoses in this cohort.

An increased HR is another way to improve the efficiency of gas exchange in the lungs and periphery, which might explain the clearer pattern we observed for this variable (Figure 2). However, higher HRs during exercise can furthermore easily be explained by a lack of training.^41^^,^^42^ As horses presented with modEA and sEA were less likely to be frequently trained compared to the healthy and miEA group, we cannot exclude this confounder, which is a typical limitation observed in studies comparing leisure horses of different training levels. Our finding is in line with other studies which found good associations between lower respiratory disease and increased HRs in poor-performing sport horses,^43–45^ as well as in asthmatic horses compared to healthy controls.^37^ It remains uncertain whether training status had an influence on the more homogenous study groups in those studies. Afterall, this variable also did not yield a diagnostic benefit due to the large overlap in HR ranges.

Beyond that, the lack of association between diagnosis and duration of recovery (for HR and RR) is another important finding of our study. An anxious healthy Icelandic horse with high HR and RR might have contributed to this lack of significance, but it remains that there is a large overlap between phenotypes and wide intra-group variations (Figure 4). Observing short recovery times (<20 minutes) in horses of all phenotypes could indicate either the influence of an optimal training status or positive effects of exercise, such as exercise-induced bronchodilation, in individual asthmatic horses.^46^ The usefulness of prolonged HR or RR recovery as indicator for respiratory disease has been discussed with differing results elsewhere.^37^^,^^44^^,^^47^^,^^48^ Based on our data, we suggest that increased recovery times could be useful as a rule-in criterion for asthma; however, short recovery times can not rule it out.

### Arterial blood gas and lactate analysis

In contrast to arterial blood gas analysis, blood lactate has become a readily available variable to assess fitness in field settings with point-of-care devices.^12^^,^^24^ Given that lactate is related to anaerobic metabolism, one could hypothesize that lactate accumulation during exercise should significantly increase in horses with asthma exhibiting marked hypoxemia. However, this effect was probably not strong enough to affect lactate accumulation (Figure 2), and no meaningful patterns were observed during exercise.

Repeated sampling of the common carotid artery is associated with a higher risk for hematoma, especially in exercising horses with high arterial blood pressures.^49^ To overcome this risk, we used an arterial catheter in the transverse facial artery. While the procedure was successful in the majority of horses, it is limited in its clinical applicability and exclusive to clinical settings with a blood gas analyzer close by. From all time points, only basal pO_2_ (T0) had a strong association with diagnosis (Figure 2). This effect is, however, attributable to the sEA group. The distinction of sEA from all other diagnoses for basal pO_2_ was possible with good accuracy because less severe asthma is rarely associated with pO_2_ <85 mm Hg at rest (Figure 3). The association with diagnosis was also observed at the end of recovery, but less pronounced (Figure 2, T7). Directly after exercise, mild and physiological exercise-induced hypoxemia was observed in healthy and miEA horses (T0 to T6, Figure 2). This finding is in line with other studies and contributes to the observed overlaps of phenotypes at T6. ^6^^,^^50^ All healthy and miEA horses showed a normalization of pO_2_ within the time it took for them to recover clinically, while the pattern was more heterogenous for the other phenotypes (Figure 5). Interestingly, some horses with sEA had similar or even higher pO_2_ at the end of exercise compared to basal values (Figure 5, Supplementary File 4), suggesting that exercise might have contributed to improved oxygenation in those individuals. This might be related to compensatory mechanisms such as the higher HRs or exercise-induced bronchodilation, decreasing the effects of (eg, lactate production) and hypoxemia itself.^46^ Variance within phenotypes suggests that clinically asthmatic horses of this cohort exhibit a degree of variability in their lung capacity, for example, based on their extent of remodeling of lung parenchyma. However, further lung function testing to characterize such differences was not in the scope of this study.

Overall, our results are consistent with those of other studies that recommend solely resting pO_2_ for the staging of asthma,^51^ while displaying the limited benefit of pO_2_ during and after exercise. The lack of strong association between diagnoses and even combinations of variables is further illustrated in a global analysis of the dataset (Figure 6).

### Sources of variability and main limitations

Data of the present study originate from a standardized examination protocol offered to owners of a clinic cohort as health check-up. The sample size was originally calculated a priori for a prospective study with a different primary outcome,^52^ which results in unequal and limited sample size. Furthermore, the SET was performed under field conditions which are heterogenous by nature. External sources of variation such as the weather and season were not controlled for. Changes in humidity, outdoor temperature, dust exposure, or a combination of these modalities likely increased phenotypical variability.^17^^,^^53^ Individual factors comprise differences in breed, temperament, BCS, and training level, which result from convenience sampling of a clinical cohort. While statistically more meaningful patterns might have emerged from a larger cohort, or fewer missing data points in the blood gas data, we believe that the heterogeneity of the described phenotypes illustrates the lack of diagnostic potential of a SET in the field.

### Clinical applicability and diagnostic performance of the SET

The presented SET variables showed only weak associations with the asthma phenotypes, and no single variable was sufficient for ruling asthma in or out. There was a considerable overlap and intra-group variability across phenotypes even with multivariable analysis. Resting arterial blood gases can aid in identifying sEA, but a definitive diagnosis of asthma requires comprehensive examinations, including endoscopy and BALF cytology.

## Supplementary Material

Supplementary_material_aalag227

## Data Availability

Additional dataset available on request from the corresponding author.

## Off-label antimicrobial declaration

Authors declare no off-label use of antimicrobials.

## Institutional animal care and use committee or other approval declaration

This study did not require official or institutional ethical approval, since all examinations were part of diagnostics and were performed at the owners’ demand. However, the standardized examination protocol was evaluated in association with a study registered and approved by the State Office for Consumer Protection and Food Safety (LAVES) in accordance with the German Animal Welfare Law under the Reference: 33.9-42502-05-21A626.

## Appendix: Material and methods supplementary information

### Study design

A standardized health and performance check-up for privately owned horses, including the SET, was offered at the Clinic for Horses of the University of Veterinary Medicine Hannover, Foundation. None of the horses received medical treatment 14 days before presentation.

Before the start of the SET, a venous catheter (18 G Milacath®, Mila International, Inc., Florence, Kentucky 41042, United States) was inserted into the *V. jugularis externa* under local anesthesia with 1.5 mL lidocaine (2%, bela-pharm GmbH, Vechta, Germany). PO_2_ and pCO_2_ were analyzed at rest (T0) and at 3 defined time points of the SET after stopping the horses (see Figure 1). In total, the SET included 6 exercise phases and the individual recovery period. The first sampling time point during the SET (T3) followed 3 of the exercise phases (2 minutes trot, 2 minutes trot and 2 minutes gallop, respectively), and the second sampling time point during the SET (T6) followed 3 additional exercise phases (another 2 minutes gallop, 3 minutes trot and 3 minutes gallop, respectively). The last sampling followed HR and RR recovery (T7). A sterile arterial catheter was positioned in the transverse facial artery (*Arteria (A.) transversa faciei*; 20/22 G Easy Flow I.V. Catheter®, WDT, Garbsen, Germany). For the placement of the catheter, local anesthesia was performed with 0.5 mL lidocaine. All horses were additionally restrained with a nose twitch. The arterial catheter was secured with 2 single stitches and shielded with gauze and adhesive padding material beneath an elastic face mask. In the event of unsuccessful catheter placement, arterial blood was obtained via puncture of the common carotid artery (*Arteria (A.) carotis communis*). In these cases, the third time point (T3) of the SET was disregarded due to concerns regarding the safety of repeated punctures.

For recording and sampling, the horses were brought to a halt at the lunge as quickly as feasible. The HR and RR were recorded by a veterinarian or veterinary student immediately after the stop of the horse. RR was recorded before HR. Arterial blood (2 mL) was obtained and transferred into electrolyte-balanced heparinized plastic syringes (PICO50®, Radiometer GmbH, Krefeld, Germany). The samples were analyzed within 10 minutes of sampling (ABL825 Flex®, Radiometer GmbH, Krefeld, Germany). Venous blood for lactate measurements was collected into sodium fluoride-balanced tubes (Vacuette®, Greiner Bio-One GmbH, Frickenhausen, Germany). Lactate was measured within 3 hours.

### Statistical analysis

Statistical analysis was performed with R 4.5.^27^ The association between variables (HR, RR, lactate, pO_2_, pCO_2_, and AaDO_2_ during recovery as well as duration of recovery) and diagnosis was analyzed with ordinal regression with 1 model per time point. This relied on the clm function from the ordinal R package. Separate analysis of defined time points, rather than continuous measures over time, were chosen to assess the feasibility of sampling at defined time points during a SET in a clinical setting. *P-*values were adjusted for multiple comparisons across all predictors and time points with the Benjamini-Hochberg procedure.^54^ Kendall’s Tau B was additionally used as indicator of effect size. The association of the length of HR and RR recovery with EA diagnosis was analyzed with ordinal models as well. The resulting *P*-values were adjusted for multiple comparisons with the Holm method.^55^ For selected significant predictors, the predictive value of the model was estimated. Please note that due to the small sample size, out-of-bag estimates could not be obtained.

To avoid assuming a similar time course of recovery between animals, we also investigated extreme values within each horse (HR, RR, pCO_2_, and AaDO_2_ maxima as well as minimum pO_2_ throughout all time points) in a similar fashion. Other correlations were described with Spearman’s rho.

The baseline values for lactate, pCO_2_, pO_2_, AaDO_2_, HR, and RR, as well as their relative values over time during exercise and recovery, were consolidated into a single dataset for multivariate analysis using principal component analysis. Since duration of recovery and intervals of measurement vary individually, only time points of recovery available in >50% of the horses were kept. Missing values were estimated with the k-nearest neighbor algorithm.

## Abbreviations

AaDO_2_: alveolar-arterial oxygen gradient
art. BGA: arterial blood gas analysis
AST: aspartate aminotransferase
BALF: bronchoalveolar lavage fluid
CI: confidence interval
CK: creatine kinase
EA: equine asthma
ECG: electrocardiography
HR: heart rate
LDH: lactate dehydrogenase
OR: odds ratio
pCO_2_: arterial carbon dioxide partial pressure, pO_2,_ arterial oxygen partial pressure
RR: respiratory rate
