# Supplementary material for: Association of submaximal exercise test variables with diagnosis of asthma in a mixed-breed cohort of leisure horses
Source: J Vet Intern Med. 2026 Sep 22;40(5):aalag227. doi: 10.1093/jvimsj/aalag227 (PMC13594658; doi:10.1093/jvimsj/aalag227)
Supplement: Supplementary file 1 [file supplementary_material_aalag227.zip › Supplementary_File_4.pdf]

Supplementary File 4: Arterial oxygen (pO_2) and carbon dioxide (pCO_2) partial pressure at baseline (time point (T) 0), during the SET (T3), directly after exercise (T6) and after individual recovery (T7).

**pCO_2 (mmHg)**				
**Horse ID**	**basal (T0)**	**T3**	**T6**	**T7**
1	46.10	41.29	48.20	47.80
2	40.90	40.60	42.30	39.80
3	39.90	40.40	36.90	38.60
4	38.60			36.70
5	54.20	53.80	54.30	44.40
6	44.10	41.50	42.70	43.80
7	40.50		43.00	
8	40.40	42.40	42.10	34.50
9	45.20		38.50	40.20
10	41.80	40.10	40.30	39.20
11	43.39	46.99	44.40	44.10
12	38.50			36.70
13	39.70	42.00	45.20	40.20
14	44.60		41.70	43.70
15	39.30	43.30	41.40	38.59
16	42.80	41.70	36.70	40.60
17	42.20	48.60	47.00	45.00
18	42.60		46.70	39.70
19	43.10	43.40	44.50	42.00
20	43.80		44.20	41.70
21	45.10		45.20	45.10
22	43.80		45.10	45.20
23	43.20	38.90	39.90	42.10
24	49.10	47.80	51.50	47.60
25	45.00	43.70	44.80	43.60
26	41.80	42.70	43.50	37.50
**pO_2 (mmHg)**				
1	96.00	89.60	67.80	81.80
2	79.10	74.90	70.50	88.00
3	84.30	74.40	88.50	78.70
4	112.00			102.00
5	67.00	64.40	65.60	69.60
6	80.90	98.00	83.00	79.10
7	84.50		80.10	

8	104.00	77.60	80.80	105.00
9	82.45		93.20	88.50
10	101.00	35.00	80.00	101.00
11	105.00	70.60	72.30	104.00
12	102.00			99.40
13	103.00	82.20	66.50	93.50
14	94.10		89.30	93.60
15	105.00	86.00	82.20	100.00
16	104.00	94.60	103.00	93.20
17	105.00	67.40	67.30	95.30
18	92.20		76.60	96.00
19	99.40	83.70	73.20	93.30
20	99.60		78.60	92.60
21	105.00		76.30	104.00
22	97.50		75.70	94.00
23	94.90	101.00	76.20	80.00
24	88.20	81.90	57.30	101.00
25	95.50	93.60	76.00	106.00
26	101.00	78.80	70.10	113.00
