# Supplementary material for: Association of submaximal exercise test variables with diagnosis of asthma in a mixed-breed cohort of leisure horses
Source: J Vet Intern Med. 2026 Sep 22;40(5):aalag227. doi: 10.1093/jvimsj/aalag227 (PMC13594658; doi:10.1093/jvimsj/aalag227)

Supplementary File 1: Muscle enzymes (aspartate aminotransferase (AST), creatine kinase (CK) and lactate dehydrogenase (LDH)) were measured before exercise (0) and at 4-, 6- and 24-hours post-exercise. The dotted line indicates the upper laboratory reference, if within the displayed range.

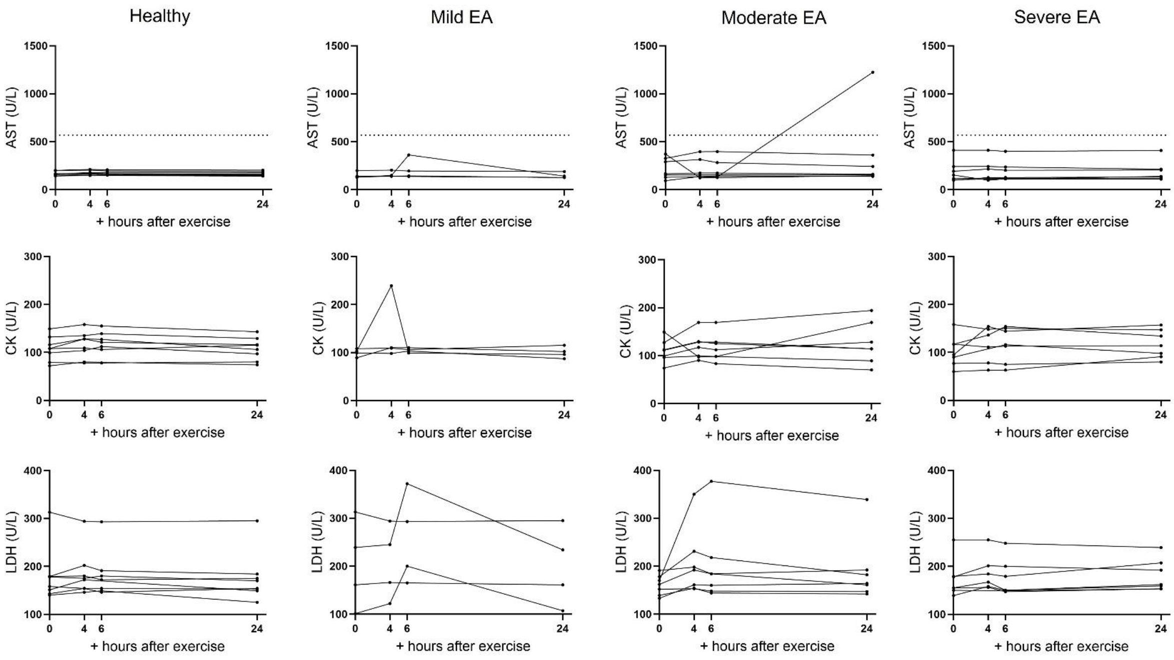

Supplement: Supplementary file 1 [file supplementary_material_aalag227.zip › Supplementary_File_1.pdf]
