# Supplementary material for: Association of submaximal exercise test variables with diagnosis of asthma in a mixed-breed cohort of leisure horses
Source: J Vet Intern Med. 2026 Sep 22;40(5):aalag227. doi: 10.1093/jvimsj/aalag227 (PMC13594658; doi:10.1093/jvimsj/aalag227)
Supplement: Supplementary file 1 [file supplementary_material_aalag227.zip › Supplementary_File_2.pdf]

Supplementary File 2: Median and quartiles [25%; 75%] of data presented in Fig. 2 for each diagnosis and time point.

Parameter	Diagnosis	Time point	1. Quartil	Median	3. Quartil
Heart rate (bpm)	healthy	0	32	36	36
Heart rate (bpm)	healthy	1	44	48	52
Heart rate (bpm)	healthy	2	44	48	52
Heart rate (bpm)	healthy	3	52	60	60
Heart rate (bpm)	healthy	4	52	68	84
Heart rate (bpm)	healthy	5	60	60	68
Heart rate (bpm)	healthy	6	64	72	80
Heart rate (bpm)	healthy	7	36	36	38
Heart rate (bpm)	miEA	0	32	32	36
Heart rate (bpm)	miEA	1	40	44	44
Heart rate (bpm)	miEA	2	40	44	44
Heart rate (bpm)	miEA	3	44	52	56
Heart rate (bpm)	miEA	4	48	60	64
Heart rate (bpm)	miEA	5	52	58	60
Heart rate (bpm)	miEA	6	60	60	64
Heart rate (bpm)	miEA	7	36	36	36
Heart rate (bpm)	modEA	0	36	36	36
Heart rate (bpm)	modEA	1	48	48	48
Heart rate (bpm)	modEA	2	48	52	52
Heart rate (bpm)	modEA	3	52	64	68
Heart rate (bpm)	modEA	4	68	80	80
Heart rate (bpm)	modEA	5	64	64	68
Heart rate (bpm)	modEA	6	70	80	84
Heart rate (bpm)	modEA	7	36	40	40
Heart rate (bpm)	sEA	0	36	36	40
Heart rate (bpm)	sEA	1	44	60	68
Heart rate (bpm)	sEA	2	44	64	64
Heart rate (bpm)	sEA	3	64	96	100
Heart rate (bpm)	sEA	4	76	84	84
Heart rate (bpm)	sEA	5	72	76	84
Heart rate (bpm)	sEA	6	60	88	116
Heart rate (bpm)	sEA	7	40	40	40

Respiratory rate (rpm)	healthy	0	12	12	16
Respiratory rate (rpm)	healthy	1	16	20	28
Respiratory rate (rpm)	healthy	2	16	24	36
Respiratory rate (rpm)	healthy	3	32	36	48
Respiratory rate (rpm)	healthy	4	36	44	64
Respiratory rate (rpm)	healthy	5	36	40	56
Respiratory rate (rpm)	healthy	6	52	66	80
Respiratory rate (rpm)	healthy	7	16	16	16
Respiratory rate (rpm)	miEA	0	10	12	12
Respiratory rate (rpm)	miEA	1	16	16	20
Respiratory rate (rpm)	miEA	2	16	20	24
Respiratory rate (rpm)	miEA	3	20	20	48
Respiratory rate (rpm)	miEA	4	20	20	64
Respiratory rate (rpm)	miEA	5	20	24	68
Respiratory rate (rpm)	miEA	6	28	52	76
Respiratory rate (rpm)	miEA	7	12	16	18
Respiratory rate (rpm)	modEA	0	12	16	16
Respiratory rate (rpm)	modEA	1	20	24	24
Respiratory rate (rpm)	modEA	2	24	28	40
Respiratory rate (rpm)	modEA	3	28	32	44
Respiratory rate (rpm)	modEA	4	32	40	60
Respiratory rate (rpm)	modEA	5	44	52	52
Respiratory rate (rpm)	modEA	6	52	68	72
Respiratory rate (rpm)	modEA	7	16	16	16
Respiratory rate (rpm)	sEA	0	16	20	20
Respiratory rate (rpm)	sEA	1	24	32	36
Respiratory rate (rpm)	sEA	2	32	40	40
Respiratory rate (rpm)	sEA	3	36	40	48
Respiratory rate (rpm)	sEA	4	40	44	48
Respiratory rate (rpm)	sEA	5	36	40	40
Respiratory rate (rpm)	sEA	6	44	52	60
Respiratory rate (rpm)	sEA	7	16	16	20
Lactate (mmol/L)	healthy	0	0.633	0.688	0.899
Lactate (mmol/L)	healthy	2	0.344	0.488	0.655
Lactate (mmol/L)	healthy	4	0.611	0.888	1.221
Lactate (mmol/L)	healthy	6	0.611	0.699	0.999
Lactate (mmol/L)	healthy	7	0.566	0.666	0.666

Lactate (mmol/L)	miEA	0	0.777	0.877	0.955
Lactate (mmol/L)	miEA	2	0.322	0.466	0.644
Lactate (mmol/L)	miEA	4	0.455	0.699	0.844
Lactate (mmol/L)	miEA	6	0.455	0.644	0.688
Lactate (mmol/L)	miEA	7	0.455	0.644	0.655
Lactate (mmol/L)	modEA	0	0.488	0.76	0.81
Lactate (mmol/L)	modEA	2	0.488	0.511	0.611
Lactate (mmol/L)	modEA	4	0.64	0.844	1.743
Lactate (mmol/L)	modEA	6	0.46	0.921	1.088
Lactate (mmol/L)	modEA	7	0.611	0.855	1.099
Lactate (mmol/L)	sEA	0	0.6	0.7	0.93
Lactate (mmol/L)	sEA	2	0.41	0.622	0.644
Lactate (mmol/L)	sEA	4	0.51	0.966	0.999
Lactate (mmol/L)	sEA	6	0.51	1.021	1.043
Lactate (mmol/L)	sEA	7	0.52	0.66	0.699
$AaDO_2$ (mmHg)	healthy	0	-10.52	-3.54	-2.52
$AaDO_2$ (mmHg)	healthy	3	0.105	1.51	11.78
$AaDO_2$ (mmHg)	healthy	6	16.93	17.73	20.91
$AaDO_2$ (mmHg)	healthy	7	-10.65	-0.77	1.86
$AaDO_2$ (mmHg)	miEA	0	-9.51	-8.02	-4.62
$AaDO_2$ (mmHg)	miEA	3	15.03	20.39	20.4
$AaDO_2$ (mmHg)	miEA	6	15.88	21.93	23.68
$AaDO_2$ (mmHg)	miEA	7	-9.4	-1.83	5.01
$AaDO_2$ (mmHg)	modEA	0	-4.77	-0.4	-0.12
$AaDO_2$ (mmHg)	modEA	3	3.01	8.08	8.52
$AaDO_2$ (mmHg)	modEA	6	8.31	14.76	16.31
$AaDO_2$ (mmHg)	modEA	7	1.51	4.11	4.46
$AaDO_2$ (mmHg)	sEA	0	10.78	14.61	14.98
$AaDO_2$ (mmHg)	sEA	3	-0.15	18.08	24.83
$AaDO_2$ (mmHg)	sEA	6	13.36	15.88	16.26
$AaDO_2$ (mmHg)	sEA	7	10.98	11.98	15.88
pCO_2 (mmHg) (mmHg)	healthy	0	39.3	43.1	43.81
pCO_2 (mmHg)	healthy	3	38.9	42.7	43.4
pCO_2 (mmHg)	healthy	6	41.4	44.51	44.81
pCO_2 (mmHg)	healthy	7	37.5	42.01	43.61
pCO_2 (mmHg)	miEA	0	39.7	42.2	43.39
pCO_2 (mmHg)	miEA	3	42	46.99	46.99

pCO_2 (mmHg)	miEA	6	44.2	44.4	45.2
pCO_2 (mmHg)	miEA	7	40.2	41.7	44.1
pCO_2 (mmHg)	modEA	0	40.4	42.8	44.6
pCO_2 (mmHg)	modEA	3	41.29	41.71	42.4
pCO_2 (mmHg)	modEA	6	41.7	42.1	46.7
pCO_2 (mmHg)	modEA	7	36.7	40.6	43.7
pCO_2 (mmHg)	sEA	0	40.5	41.8	44.1
pCO_2 (mmHg)	sEA	3	40.1	40.4	41.5
pCO_2 (mmHg)	sEA	6	38.5	42.3	42.7
pCO_2 (mmHg)	sEA	7	39.2	39.8	40.21
pO_2 (mmHg)	healthy	0	95.5	99.4	105
pO_2 (mmHg)	healthy	3	78.8	83.7	93.6
pO_2 (mmHg)	healthy	6	73.2	76	76.2
pO_2 (mmHg)	healthy	7	93.3	100	104
pO_2 (mmHg)	miEA	0	99.6	103	105
pO_2 (mmHg)	miEA	3	67.4	70.6	70.6
pO_2 (mmHg)	miEA	6	66.5	67.3	72.3
pO_2 (mmHg)	miEA	7	92.6	93.5	95.3
pO_2 (mmHg)	modEA	0	92.2	96	102
pO_2 (mmHg)	modEA	3	77.6	81.9	89.6
pO_2 (mmHg)	modEA	6	67.8	76.6	80.8
pO_2 (mmHg)	modEA	7	93.2	96	99.4
pO_2 (mmHg)	sEA	0	79.1	82.45	84.3
pO_2 (mmHg)	sEA	3	35.01	64.4	74.9
pO_2 (mmHg)	sEA	6	70.5	80.1	83
pO_2 (mmHg)	sEA	7	78.7	79.1	88
