# Supplementary material for: Association of submaximal exercise test variables with diagnosis of asthma in a mixed-breed cohort of leisure horses
Source: J Vet Intern Med. 2026 Sep 22;40(5):aalag227. doi: 10.1093/jvimsj/aalag227 (PMC13594658; doi:10.1093/jvimsj/aalag227)

Supplementary File 3: Spearman's correlation matrix for all SET parameters. Note that age was added as clinical parameter in this analysis. Correlations of variables with diagnoses were overall weak. Abbr.: pO_2 = arterial oxygen partial pressure, $AaDO_2$ = Alveolar-arterial oxygen gradient, pCO_2 = arterial carbon dioxide partial pressure

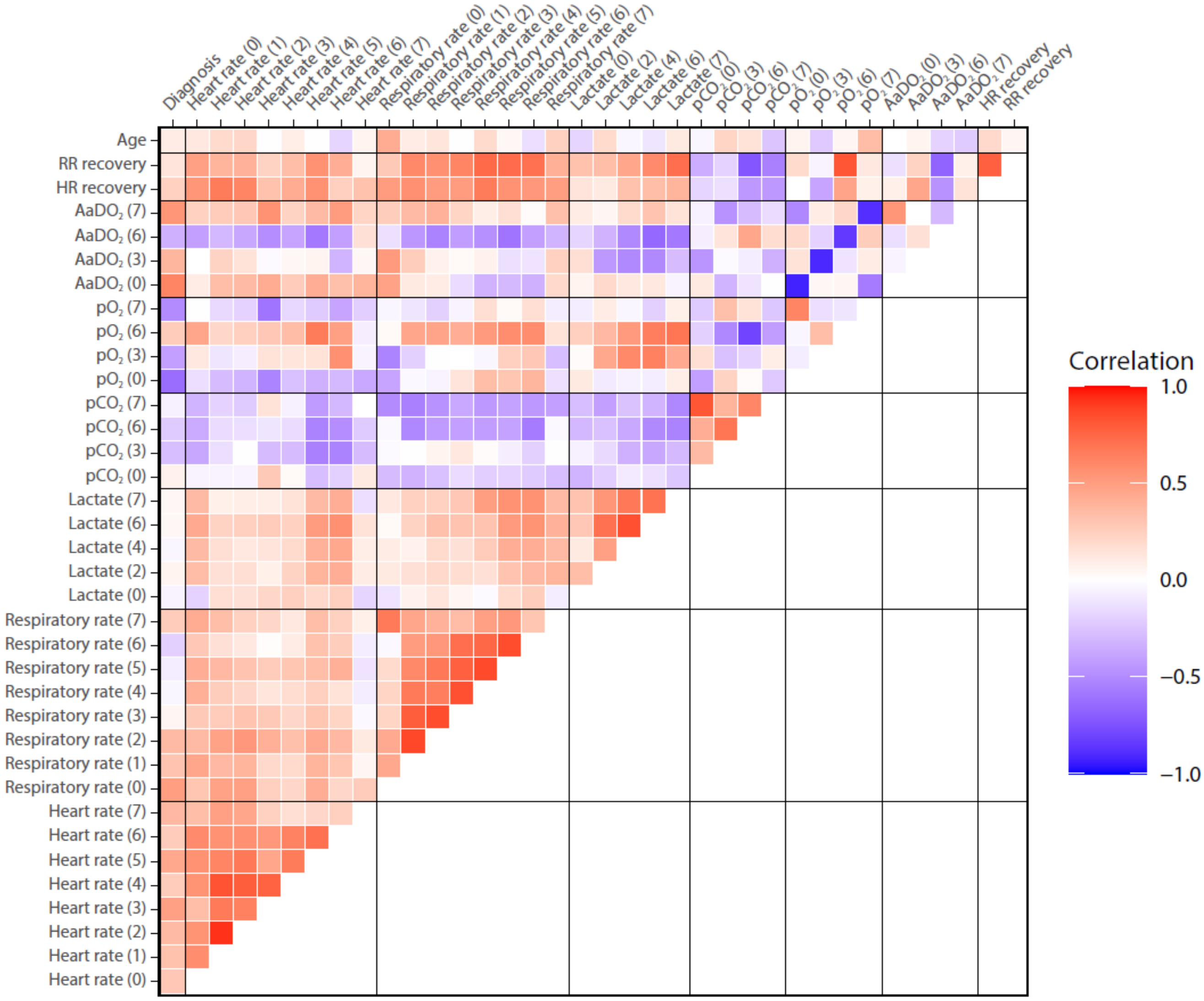

Supplement: Supplementary file 1 [file supplementary_material_aalag227.zip › Supplementary_File_3.pdf]
